# The enhancement of astaxanthin production in *Phaffia rhodozyma* through a synergistic melatonin treatment and zinc finger transcription factor gene overexpression

**DOI:** 10.3389/fmicb.2024.1367084

**Published:** 2024-04-11

**Authors:** Jianping Jia, Zhitao Chen, Qingqing Li, Feifei Li, Siru Liu, Guoliang Bao

**Affiliations:** ^1^School of Phamacy, School of Food Science and Engineering, Hangzhou Medical College, Hangzhou, China; ^2^Key Laboratory of Drug Safety Evaluation and Research of Zhejiang Province, Hangzhou Medical College, Hangzhou, China; ^3^School of Public Health, Hangzhou Medical College, Hangzhou, China

**Keywords:** *Phaffia rhodozyma*, astaxanthin, melatonin, anti-stress, signal transduction, RNA-seq, transcription factor engineering

## Abstract

Astaxanthin has multiple physiological functions and is applied widely. The yeast *Phaffia rhodozyma* is an ideal source of microbial astaxanthin. However, the stress conditions beneficial for astaxanthin synthesis often inhibit cell growth, leading to low productivity of astaxanthin in this yeast. In this study, 1 mg/L melatonin (MT) could increase the biomass, astaxanthin content, and yield in *P. rhodozyma* by 21.9, 93.9, and 139.1%, reaching 6.9 g/L, 0.3 mg/g DCW, and 2.2 mg/L, respectively. An RNA-seq-based transcriptomic analysis showed that MT could disturb the transcriptomic profile of *P. rhodozyma* cell. Furthermore, differentially expressed gene (DEG) analysis show that the genes induced or inhibited significantly by MT were mainly involved in astaxanthin synthesis, metabolite metabolism, substrate transportation, anti-stress, signal transduction, and transcription factor. A mechanism of MT regulating astaxanthin synthesis was proposed in this study. The mechanism is that MT entering the cell interacts with components of various signaling pathways or directly regulates their transcription levels. The altered signals are then transmitted to the transcription factors, which can regulate the expressions of a series of downstream genes as the DEGs. A zinc finger transcription factor gene (*ZFTF*), one of the most upregulated DEGs, induced by MT was selected to be overexpressed in *P. rhodozyma*. It was found that the biomass and astaxanthin synthesis of the transformant were further increased compared with those in MT-treatment condition. Combining MT-treatment and *ZFTF* overexpression in *P. rhodozyma*, the biomass, astaxanthin content, and yield were 8.6 g/L, 0.6 mg/g DCW, and 4.8 mg/L and increased by 52.1, 233.3, and 399.7% than those in the WT strain under MT-free condition. In this study, the synthesis and regulation theory of astaxanthin is deepened, and an efficient dual strategy for industrial production of microbial astaxanthin is proposed.

## Introduction

Astaxanthin, known as 3,3′- dihydroxy- β, β’- carotene-4,4′- dione, is a keto carotenoid known for its remarkable antioxidant, anticancer, and immune-boosting properties and widely applied in the food, aquaculture, cosmetics, and healthcare industries (Naguib, 2000; Hussein et al., 2006). Currently, astaxanthin production primarily relies on chemical synthesis and biosynthesis (Li et al., 2023). The two prominent isomers of biosynthetic astaxanthin are 3S,3S’ and 3R,3R’, which have distinct advantages over their synthetic counterparts in terms of antioxidant efficacy, stability, and bioavailability (Nutakor et al., 2022). For biosynthesis, the yeast *Phaffia rhodozyma* (also known as *Xanthophyllomyces dendrorhous*) is an ideal source of astaxanthin and has served as the prime candidate for astaxanthin production by microbial fermentation. Within the *P. rhodozyma*, the astaxanthin synthesis pathway has been meticulously elucidated. The pathway involves a 7-step transformation from acetyl-CoA and catalyzes by enzymes such as acetoacetyl-CoA transferase (ACAT), HMG-CoA synthase, HMG-CoA reductase, mevalonate kinase (MK), phosphomevalonate kinase (PMK), diphosphate decarboxylase (PMD), and isopentenyl diphosphate isomerase (IDI), leading to the production of isopentenyl-5-pyrophosphate (IPP) and dimethylallyl pyrophosphoric acid (DMAPP). Subsequently, IPP and DMAPP undergo a 4-step conversion catalyzed by geranyl pyrophosphate (GGPP) synthase (crtE), phytoene synthase (crtYB), phytoene desaturase (crtI), and lycopene cyclase (crtYB), resulting in β-carotene. Finally, astaxanthin is produced by the oxidation of β-carotene and catalyzed by astaxanthin synthase (crtS) (Kizer et al., 2008). The provision of essential elements such as Acetyl-CoA, NADPH, ATP, and Mg^2+^ is crucial for facilitating astaxanthin synthesis in *P. rhodozyma* (Basiony et al., 2022). Moreover, *P. rhodozyma* can efficiently utilize a variety of cost-effective sugars as carbon sources to achieve swift heterotrophic growth and facilitate high-density fermentation, rendering an exceptionally well-suited microbial strain for large-scale astaxanthin production (Ramirez et al., 2000; Igreja et al., 2021). Nevertheless, the relatively modest astaxanthin productivity in *P. rhodozyma* has presented a challenge to the advancement of the biosynthetic astaxanthin industry.

Currently, comprehensive strategies and the associated regulatory mechanisms governing astaxanthin synthesis in *P. rhodozyma* have been established and elucidated. Various stresses, such as high-intensity light, nitrogen starvation, oxidative stress, and other environmental stress conditions, could induce astaxanthin accumulation at the expense of cell growth, leading to low productivity. Thus, the utilization of fermentation promoters to stimulate astaxanthin production in *P. rhodozyma* has become an ideal strategy (Zhang et al., 2020a). Given the initial isolation of *P. rhodozyma* from plant materials and its evolutionary adaptation to metabolites from symbiotic plants, various plant extracts have been leveraged to enhance growth and astaxanthin accumulation in *P. rhodozyma* (Bellora et al., 2016). To date, the plant extracts from oak, maple, carrot, *Perilla frutescens*, *Allium fistulosum*, and others were found to stimulate astaxanthin synthesis in *P. rhodozyma* to varying degrees (Kim et al., 2007; Stachowiak, 2012; Kothari et al., 2019). Similarly, phytohormones have emerged as potent contributors to enhanced growth and astaxanthin accumulation in *P. rhodozyma*. Even at a concentration as low as 0.25 mg/L, 6-benzylaminopurin (6-BAP) can boost both biomass and astaxanthin production in *P. rhodozyma* by more than 20% (Pan et al., 2020). Furthermore, a high concentration of 500 mg/L gibberellic acid (GA) has been shown to elevate astaxanthin biosynthesis by 77% in a fed-batch system (Liu et al., 2022). Additionally, the introduction of 9% (v/v) n-hexadecane has the capacity to enhance oxygen transfer rates by 90%, consequently promoting astaxanthin synthesis by 57.6% (Liu and Wu, 2006). Furthermore, hydrogen peroxide (H_2_O_2_) has been found to be involved in the oxidative stress response of yeast to H_2_O_2_, resulting in a notable increase in astaxanthin synthesis, reaching up to 58.3 mg/L (Liu and Wu, 2007).

Melatonin (MT), known as N-acetyl-5-methoxytryptamine, is a pivotal indole-type small molecule that plays an essential role across various life forms, such as animals, plants, fungi, and bacteria. MT functions as a hormone regulating the biological clock while simultaneously serving as a potent antioxidant. MT exhibits impressive antioxidant properties, surpassing the effectiveness of glutathione (GSH) by 5-fold in neutralizing hydroxide ions (OH^−^) and exogenous scavenger mannitol by 15-fold (Poeggeler et al., 2002). Furthermore, MT operates as a signaling molecule at the cellular level, upregulating numerous antioxidant enzymes, thereby enhancing its efficacy as an antioxidant (Shi et al., 2015). In plants, MT functions as a biostimulator exerting control over various developmental processes that promote seed germination, enhance fruit quality, and increase yield (Debnath et al., 2019). Recent advancements have revealed the potential of MT as a powerful stimulator in microalgal biotechnology, offering a potential solution to industrial challenges. In the presence of 1 μM MT treatment, *Monoraphidium* sp. QLY-1 exhibited a remarkable 1.32-fold increase in lipid content, reaching 49.6%, albeit with a concomitant decrease in protein and carbohydrate contents. These changes were correlated with altered activities of reactive oxygen species (ROS) and lipid biosynthesis-related enzymes, highlighting the pivotal role of MT in lipid accumulation (Li et al., 2017). Similarly, although MT alone did not significantly impact the growth and pigment accumulation of *Dunaliella bardawil* under low light intensities, when coupled with 9,500 LUX light intensity, it led to notable increases in lutein, α-carotene, and β-carotene contents, exceeding 20% enhancements compared with the control group (Xie et al., 2022). In *Haematococcus pluvialis*, MT was exogenously added to mitigate reactive oxygen species (ROS) bursts and limit cellular damage induced by abiotic stress. This mechanism resulted in a remarkable 2.25-fold increase in astaxanthin content, reaching 32.4 mg/g. The observed effects were associated with melatonin’s role in activating the nitric oxide (NO)-mediated mitogen-activated protein kinase (MAPK) and cyclic adenosine monophosphate (cAMP) pathways, both positively influencing astaxanthin biosynthesis (Ding et al., 2018b). However, the specific impacts of MT on *P. rhodozyma* and the underlying mechanisms governing these effects remain an area of ambiguity and warrant further investigation.

A robust genetic transformation system has been successfully established in *P. rhodozyma* and opens the door to powerful genetic modifications for the creation of high-astaxanthin-yield strains (Adrio and Veiga, 1995). However, current research efforts predominantly focus on enhancing the astaxanthin synthesis pathway to increase productivity (Hara et al., 2014; Breitenbach et al., 2019). Despite significant progress, our understanding of the global regulatory mechanisms governing astaxanthin synthesis in *P. rhodozyma* remains incomplete. Transcription factors (TFs) serve as vital global regulators within intricate signaling networks. They engage with specific DNA motifs within the cis elements of target genes or signaling molecules and transmit stress signals to the nucleus while orchestrating the transcription of related genes. Thus, transcription factor engineering (TFE) stands out as a potent tool capable of regulating the expression of multiple genes with a single genetic transformation and offers an efficient means to induce global metabolic variations (Grama et al., 2022). In the context of *P. rhodozyma*, the sterol regulatory element-binding protein1 (Sre1) represents a TF that stimulates the transcriptional expression of *CrtE* and *CrtR* within the astaxanthin biosynthesis pathway (Gómez et al., 2020). Conversely, the zinc finger protein MIG1 has been shown to inhibit the expression of *CrtI*, *CrtYB*, and *CrtS* in *P. rhodozyma* under high-sugar conditions, resulting in reduced astaxanthin accumulation (Wozniak et al., 2011). Despite of these valuable insights, the specific TFs governing astaxanthin regulation in this yeast species warrant further exploration. Additionally, the development of high-astaxanthin-producing strains through the manipulation of TFs via TFE holds considerable promise and merits further development and investigation.

In this study, we investigate the effects of MT on biomass, astaxanthin content, and yield in *P. rhodozyma*. Then, an RNA-seq-based transcriptomic analysis is performed to illuminate the molecular mechanism of MT, enhancing astaxanthin productivity in *P. rhodozyma.* A Zinc Finger TF (*ZFTF*) gene, as one of the differently expressed genes (DEGs) and induced by MT condition, is selected to be overexpressed in *P. rhodozyma* for further improving astaxanthin productivity. Thus, in this study, we present an efficient strategy in combination with chemical treatment and genetic modification to increase astaxanthin production and break the bottleneck of yeast astaxanthin productivity in *P. rhodozyma.*

## Materials and methods

### Chemicals

All reagents used in this study, including methanol, ethanol, glucose, peptone, yeast powder, astaxanthin, and MT, were purchased from Shanghai Aladdin Biochemical Technology Co., Ltd. (Shanghai, China). Astaxanthin and MT were of HPLC grade, while all other reagents were at least analytical grade standards.

### Microbial strains, medium compositions, and culture condition

The *P. rhodozyma* strain AS2.1557 was acquired from the China General Microbiological Culture Collection Center (Beijing, China). For the seed culture, the strain was cultivated on yeast extract, peptone, dextrose (YPD) medium consisting of 20 g/L glucose, 10 g/L yeast extract, and 20 g/L peptone. Alternatively, for fermentation, the strain was cultured on yeast extract (YM) medium composed of 5 g/L tryptone, 3 g/L yeast extract, 3 g/L malt extract, and 10 g/L glucose. The culture conditions were maintained at 22°C and 300 rpm for 96 h. For MT treatment, MT was dissolved in dimethyl sulfoxide (DMSO) with a concentration of 1 g/L as the stock solution, and the stock solution was added to YM medium with the final MT concentrations of 0.25 mg/L. 0.5 mg/L, 1 mg/L, 2 mg/L, and 3 mg/L, respectively. An equivalent volume of DMSO was introduced into the YM medium to serve as the control. Positive *P. rhodozyma* transformants were selected using YM medium plates supplemented with 50 mg/L G418.

### Biomass measurement

The biomass of *P. rhodozyma* AS2.1557 was quantified in terms of dry cell weight (DCW). An aliquot of 50 mL cell culture was centrifuged at 4°C and 7,000 g for 5 min and then washed twice with distilled water. Subsequently, the cell pellets were lyophilized at approximately −50°C for 48 h until a constant weight was attained (Yu et al., 2019).

### Astaxanthin extraction and analysis

In total, 3 mL of DMSO was preheated to 60°C and thoroughly mixed with the *P. rhodozyma* cells. The mixture was incubated at 50°C for 5 min. Subsequently, 3 mL of anhydrous ethanol was introduced to the mixture and incubated for 20 min. The sample underwent extraction through ultrasonication for 10 min, followed by centrifugation at 8000 rpm and 4°C for 10 min. This extraction process was repeated in several cycles until the cell pellet achieved a white coloration. The supernatants obtained from each cycle were collected and diluted to a final volume of 20 mL. The astaxanthin was analyzed on an Essentia LC-16 HPLC system equipped with a Hypersil BDS C18 column (Shimadzu, Japan). The UV detection wavelength of astaxanthin, injection volume, column temperature, and flow rate were 478 nm, 10 μL, 25°C, and 1 mL/min, respectively. The mobile phage was composed of methanol and acetonitrile with a volume ratio of 9:1 (Pan et al., 2020).

### RNA extraction and library construction

Total RNA was extracted from each sample utilizing TRIzol reagent (Invitrogen, United States). The quantity and purity of the RNA were assessed using an Agilent 2,100 Bioanalyzer (Agilent Technologies, United States), a NanoDrop apparatus (Thermo Fisher Scientific Inc., United States), and 1% agarose gel electrophoresis, respectively. The integrity of the RNA was evaluated with a Bioanalyzer 2,100 (Agilent Technologies, United States), ensuring a RIN (RNA Integrity Number) exceeding 7.0, which was further confirmed through electrophoresis with denaturing agarose gel (Yu et al., 2019). Poly(A) RNA was purified employing Dynabeads Oligo(dT)25–61,005 (Thermo Fisher, United States) and underwent two rounds of purification. Poly(A) RNA served as the template for the synthesis of the first strand cDNA using ProtoScript II Reverse Transcriptase (Invitrogen, United States). Subsequently, the second strand cDNA was synthesized with Second Strand Synthesis Enzyme Mix, which includes dACG-TP/dUTP, *E. coli* DNA polymerase I (NEB, United States), RNase H (NEB, USA), and dUTP solution (Thermo Fisher, United States). An A-base was added to each strand, followed by ligation with an adaptor containing a T-tail. Size selection of the adaptor-ligated DNA was accomplished using AMPureXP beads. After Uracil-Specific Excision Reagent (USER) enzyme (NEB, United States) treatment of the U-labeled second-stranded DNAs, the ligated products underwent PCR amplification for 10 cycles. Both primers that used in PCR-possessed sequences could anneal with the flow cell to perform bridge PCR. The resulting PCR products were purified using AxyPrep Mag PCR Clean-Up Kit (Axygen, United States), validated by an Agilent 2,100 Bioanalyzer (Agilent Technologies, United States), and quantified using a Qubit 2.0 Fluorometer (Invitrogen, United States). The average insert size was maintained at 300 ± 50 bp. Subsequently, 2 × 150 bp paired-end sequencing (PE150) was carried out on an Illumina HiSeq instrument, following the vendor’s recommended protocol (Illumina, United States).

### RNA-Seq data analysis

The data in FASTAQ format underwent initial processing using fastp software,^1^ resulting in the extraction of clean reads while removing adapters, poly-N sequences, and low-quality reads. Key quality metrics such as Q20, Q30, and GC-content were computed, and all subsequent analyses were conducted using the high-quality clean data. For read alignment to the reference genome of *P. rhodozyma* CBS6938 (Genebank No. GCA_014706385.1), the HISAT2 tool^2^ was employed. Subsequently, StringTie was utilized to estimate the expression levels of all transcripts, which were quantified as Fragments Per Kilobase of transcript per Million mapped reads (FPKM). Differentially expressed genes (DEGs) were selected based on criteria that included a fold change >1.2 or fold change <0.7, coupled with a *p*-value derived from the parametric F-test comparing nested linear models <0.05. This analysis was carried out using the R package edgeR.^3^ To gain insights into the biological significance of these DEGs, Gene Ontology (GO) enrichment analysis was performed using the R package GOseq (Li and Dewey, 2011). Additionally, to elucidate the potential pathways associated with the DEGs, KOBAS software was employed for enriching significant DEGs within the KEGG pathway^4^ (Mao et al., 2005).

### Quantitative real-time reverse transcription PCR

To further validate the expression levels of 33 selected DEGs identified from the transcriptomic data, quantitative reverse transcription polymerase chain reaction (qRT-PCR) was conducted. Gene-specific primers were meticulously designed using Primer Premier 5.0, and their details are shown in Supplementary Table S2. Total RNA extraction was followed the previously described method. The qRT-PCR experiments were carried out utilizing the CFX96 Touch qRT-PCR system (BIORAD, United States). The PCR conditions were set as follows: an initial denaturation at 98°C for 30 s, followed by 40 cycles of denaturation at 98°C for 5 s, and an annealing/extension step at 58°C for 34 s. The 18S rRNA gene was served as an internal standard. Relative quantitative variations were assessed using the ΔΔCt method (Livak and Schmittgen, 2001). Each qRT-PCR reaction was performed in triplicate, and data normalization was accomplished using the average of the internal standard. Linear correlations between the expression levels of the DEGs deduced from the transcriptomic data and those determined by RT-PCR were evaluated, with the R^2^ value serving as a measure of the goodness of fit, validating the transcriptomic results.

### Construction of a gene overexpressing vector

An overlap PCR technique was applied to construct a TF-overexpressing vector with minor modification, as described by Yu et al. (2020). Eight fragments, 18sup, Pgdp, G418, Tgdp, Padh4, ZFTF, Tact, and 18sdown, were first separately amplified with homologous oligonucleotides of the up-stream and down-stream fragments integrated by the primers, as shown in Supplementary Table S3. In the second step PCR, the eight fragments were ligated in sequence to the overexpression vector that used them as templates with an approximate molar ratio as 1:3:5:7:7:5:3:1, respectively. The vector was composed of the G418-resisent gene and the TF gene expression cassettes and integrated into the 18 s ribosomal DNA (18srDNA) locus of the *P. rhodozyma* genome through a double-cross homologous recombination (Hara et al., 2014). PCR conditions involved an initial denaturation at 98°C for 3 min, followed by 30 cycles (98°C for 10 s, 55°C for 1 min, and 72°C for 3 min), concluding with a final extension step of 10 min at 72°C.

### Genetic transformation of the TF-overexpressing vector into *Phaffia rhodozyma* cells

The transformation of *P. rhodozyma* cells was conducted through the electro-transformation method with slight modifications, as described by Niklitschek et al. (2008). The yeast cells were first cultured on YM medium until their OD_660nm_ reached 1.5. Subsequently, the cells were harvested by centrifugation at 10,000 g for 5 min, and the cell pellet was resuspended in a potassium phosphate buffer containing 50 mM potassium phosphate (pH 7) and 25 mM DTT (Dithiothreitol). The cell suspension was incubated at 21°C for 15 min and then underwent two washes with STM buffer (270 mM sucrose, 10 mM Tris HCl, and 1 mM MgCl_2_, pH 7.5). The washed cells were suspended in 500 μL of STM buffer at 4°C as the competent cells for transformation. For transformation, 60 μL of competent cells was thoroughly mixed with 10 μg of the DNA fragment. Electroporation was performed under the following conditions: 25 μF, 1,000 Ω, and 800 V, utilizing a Gene Pulser (BioRad, United States). Transformed cells were selected by cultivating them on YM medium supplemented with 50 mg/L G418.

### Validation of TF integration into genome and its expression

Genomic DNA was extracted from both the *P. rhodozyma* wild-type strain (WT) and the transformants using the TaKaRa MiniBEST Universal Genomic DNA Extraction Kit (Takara Bio, Dalian, China). Integration of TF gene into genome was performed by PCR using the extracted genome as template and Confirm-F and Confirm-R as primers (Supplementary Table S3). RT-PCR was used to validate the expression level of TF gene and performed as described above (Supplementary Table S3).

### Statistical analysis

All experiments were repeated three times, and data were expressed as mean values ± standard deviations (SD). Data were statistically analyzed by one-way ANOVA and Scheffe’s test using SPSS® 20.0 (SPSS Inc., Chicago, IL, United States).

## Results

### Enhancing astaxanthin biosynthesis by melatonin treatment

**Figure 1:**
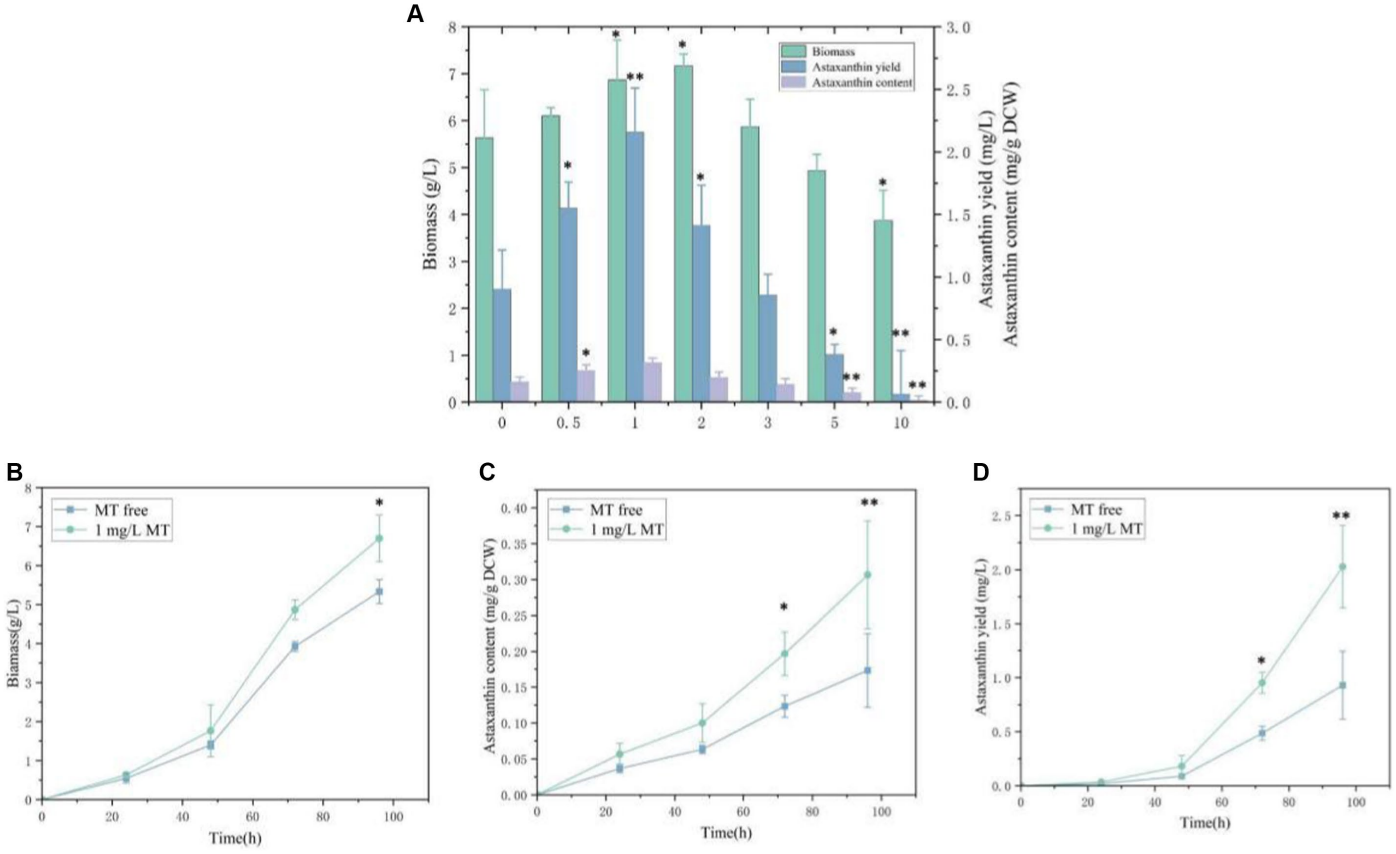
Effects of melatonin (MT) on biomass and astaxanthin accumulation in *Phaffia rhodozyma*. **(A)** Effects of different concentrations of MT on biomass, astaxanthin content, and yield in *P. rhodozyma.* **(B)** 96 h time-course effect of 1 mg/L MT on biomass in *P. rhodozyma.* **(C)** 96 h time-course effect of 1 mg/L MT on astaxanthin content in *P. rhodozyma.* **(D)** 96 h time-course effect of 1 mg/L MT on astaxanthin yield in *P. rhodozyma.* Data are given as means ± SD, *n* = 3. *p < 0.05 **p < 0.01.

To evaluate the feasibility of melatonin as a stimulating factor in the synthesis of astaxanthin in *P. rhodozyma*, the effects of 0.5 mg/L, 1 mg/L, 2 mg/L, 3 mg/L, 5 mg/L, and 10 mg/L MT on biomass, astaxanthin content, and yield were analyzed (Figure 1). A concentration range of 0.5–2 mg/L of MT had a positive effect on biomass, and the highest biomass of 7.2 g/L was obtained under the 2 mg/L MT condition with 27.2% higher than the MT-free condition. The biomass was decreased sharply when the concentration of MT was higher than 5 mg/L. Meanwhile, 1 mg/L of MT maximized astaxanthin content, reaching 0.3 mg/g DCW and 93.9% higher than the MT-free condition. However, the astaxanthin content was decreased gradually as the concentration of MT was increased from 1 mg/L. Combining biomass and astaxanthin content, astaxanthin yield reached the highest level of 2.2 mg/L with 123.1% higher than control under the 1 mg/L MT condition, which was the optimal for astaxanthin content but not for biomass. Thus, the 1 mg/L MT condition was used in the further transcriptomic study. These results indicate that *P. rhodozyma* is very sensitive to MT, and MT serves as an effective stimulator to break through the bottleneck of astaxanthin productivity.

### Sequencing data assembly and analysis

Due to the *P. rhodozyma* reference genome (NCBI number GCA_001007165.2_Xden1) has been released, we employed a transcriptome with reference strategy to illuminate the molecular mechanism of MT inducing the astaxanthin biosynthesis. The MT-free and MT-treatment samples were represented as C and M, respectively. Through data pretreatment and quality control (Supplementary Table S1), the ranges of valid ratio (valid reads to raw reads), Q20 (sequencing error rate < 0.01), and Q30 (sequencing error rate < 0.001) in the six samples were 95.19–97.67%, 99.95–99.96%, and 97.74–97.91%, respectively. These results indicated that the sequencing data had high quality and can be further analyzed. The valid reads were then mapped into the genome of *P. rhodozyma*, a range of 95.85–96.14% valid reads in all six samples could be mapped, and 6,272 genes were identified and annotated. It is confirmed that most of the genes can be identified, annotated, and further analyzed.

### Alterations in transcriptional profile of *Phaffia rhodozyma* induced by melatonin

The transcriptomic profiles of the MT-treatment and MT-free samples were compared to validate the transcriptional alterations of *P. rhodozyma* induced by MT. As shown in Supplementary Figure S1, Pearson correlation analysis showed that inter-group between the M and C groups had lower correlation, while the intra-group in the M or C group had higher correlation (Supplementary Figure S1A). Based on all transcripts, the profiles from the M and C groups could be well discriminated at the PCA 1 level, which could explain up to 99.06% difference (Supplementary Figure S1B). These results indicate that the global transcriptomic profiles of the M and the C groups can be significantly distinguished, confirming that exogenous MT can disturb the global transcriptomic profile to enhance astaxanthin biosynthesis in *P. rhodozyma*.

### Identification of DEGs, gene ontology function annotation, and KEGG pathway enrichment

**Figure 2:**
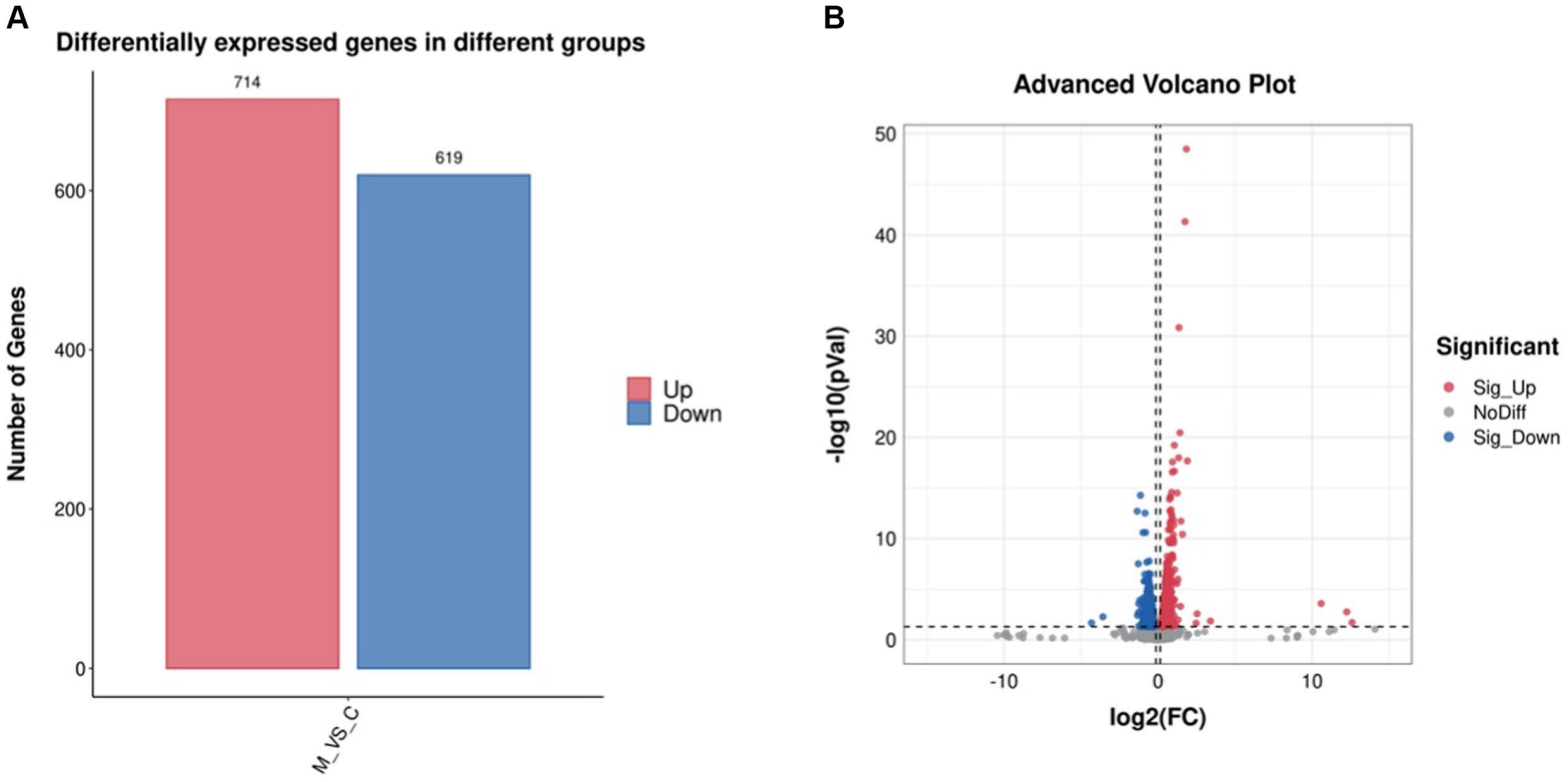
The differentially expressed gene (DEG) number **(A)** and volcano plot **(B)** induced by melatonin treatment in *P. rhodozyma.*

**Figure 3:**
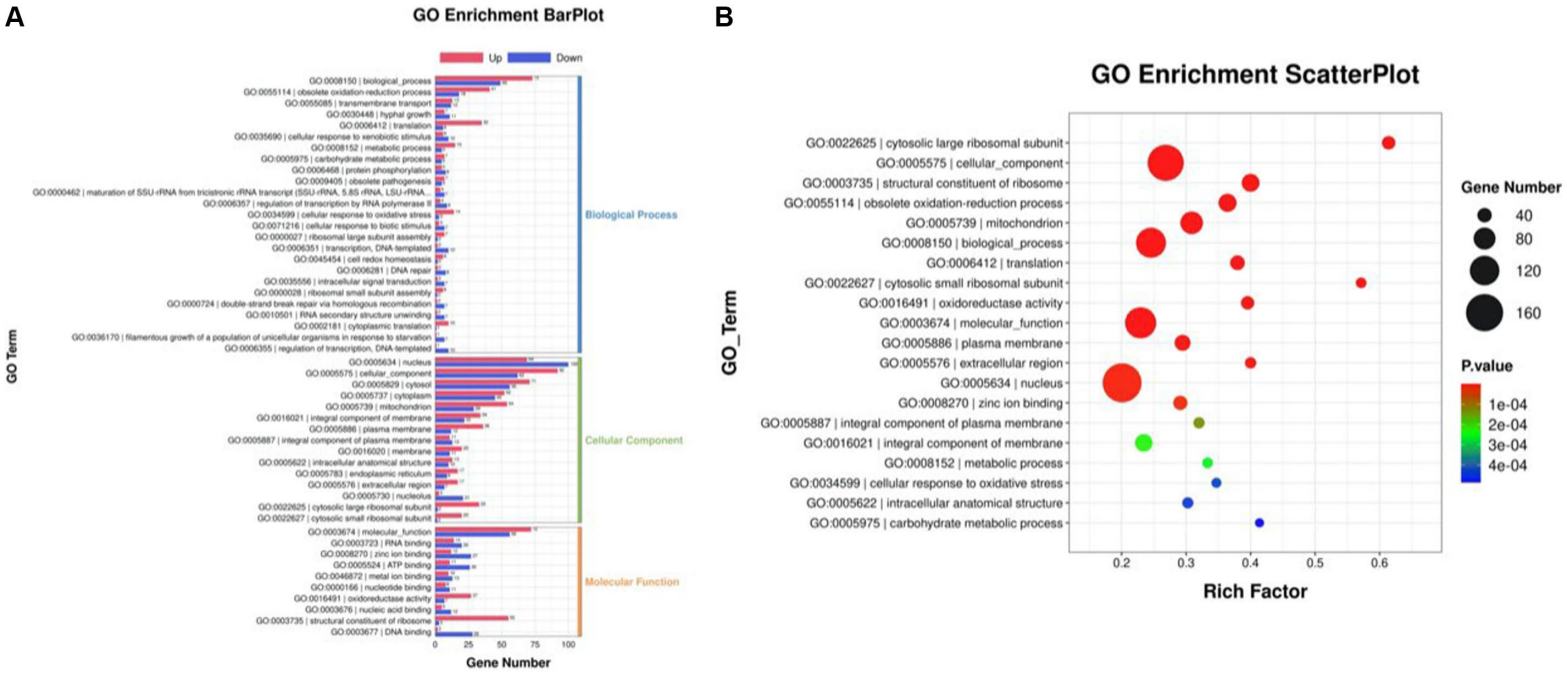
GO annotation of the DEGs between the cells from MT treatment (M) and MT-free (C) conditions. **(A)** GO enrichment BarPlot; **(B)** GO enrichment ScatterPlot.

**Figure 4:**
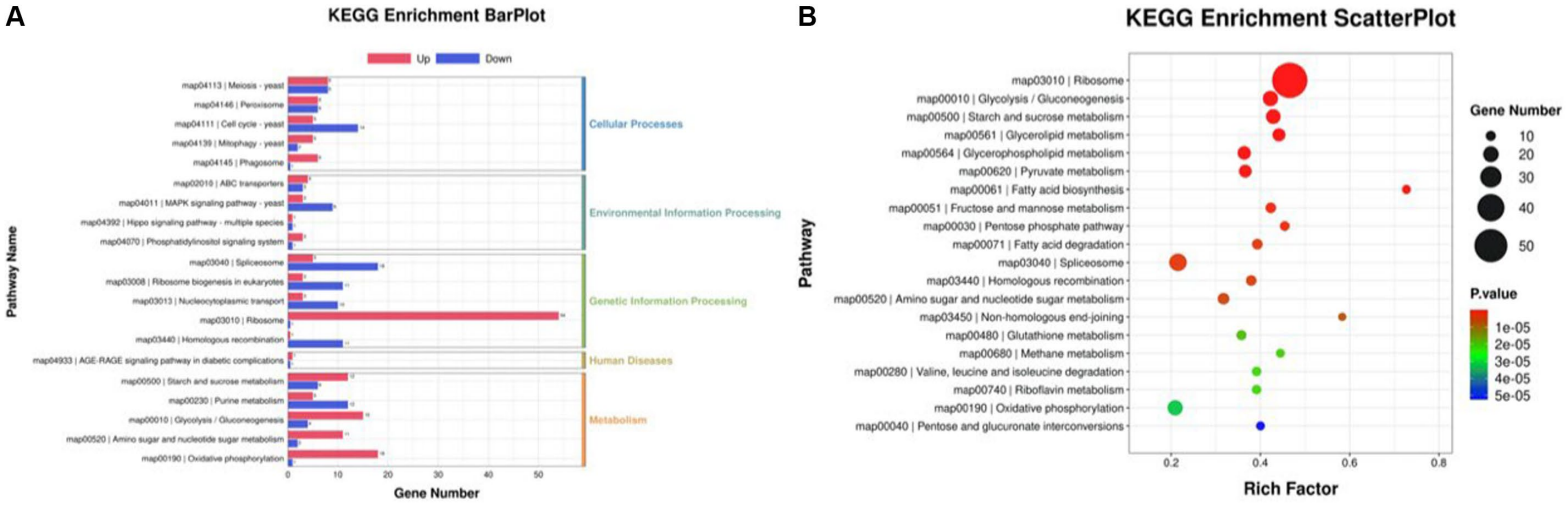
KEGG annotation of the DEGs between the cells from MT treatment (M) and MT-free (C) conditions. **(A)** KEGG enrichment BarPlot; **(B)** KEGG enrichment Scatter Plot.

The differentially expressed genes (DEGs) of the MT-treatment sample against the MT-free sample were identified, annotated, and pathway-enriched to further explain and propose the molecular mechanism of MT, enhancing astaxanthin biosynthesis in *P. rhodozyma*. A total of 1,333 DEGs with 714 upregulated and 619 downregulated genes were identified in the MT-treated *P. rhodozyma* cells against the MT-free cells (Figure 2). These DEGs were further reannotated to 50 Gene Ontology (GO) terms under 3 GO domains, namely, molecular function (MF), cellular component (CC), and biological process (BP), respectively (Figure 3A). As GO enrichment scatterplot shown in Figure 3B, the most DEGs were distributed in the “cytosolic large ribosomal subunit,” “cellular component,” “structural constituent of ribosome,” “obsolete oxidation–reduction process,” “mitochondrion,” “biological process,” “translation,” “cytosolic small ribosomal subunit,” “oxidoreductase activity,” “molecular function,” “plasma membrane,” “extracellular region,” “nucleus,” and “zinc ion binding” terms based on the gene number and enrichment factors. To identify the induced or inhibited biological pathway by MT treatment, the DEGs were enriched into the Kyoto Encyclopedia of Genes and Genomes (KEGG) and distributed in “cellular processes,” “environmental information processing,” “genetic information processing,” “human diseases,” and “metabolism” KEGG categories (Figure 4A). In “cellular processes” category, most DEGs were enriched in the “yeast cell cycle,” “yeast meiosis,” and “peroxisome” pathways. In “environmental information processing,” MAPK signaling and ABC transporters were two main pathways with 12 and 7 DEGs distributed, respectively. The “metabolism” is the most abundant category, and the DEGs were mainly enriched in “glycolysis/gluconeogenesis,” “oxidative phosphorylation,” “purine metabolism,” “starch and sucrose metabolism,” and “glycerophospholipid metabolism.” From the gene number and rich factor, the DEGs were mainly enriched in “ribosome,” “spliceosome,” “yeast cell cycle,” “glycolysis/gluconeogenesis,” and “oxidative phosphorylation” pathways (Figure 4B).

The DEGs that can explain enhancement of astaxanthin biosynthesis in *P. rhodozyma* induced by MT-treatment are presented in Table 1 from seven perspectives, including astaxanthin biosynthesis, fatty acid metabolism, sterol metabolism, substrate transportation, anti-stress, signal transduction, and transcription factor. Except for astaxanthin synthase and geranyl pyrophosphate synthase, which were increased by 0.4- and 0.3-fold induced by MT treatment, there were no significant changes in other genes in the astaxanthin synthesis pathway. For fatty acid metabolism, fatty acid-2 hydroxylase, acyl-dehydrogenase, and acetyl-c-acyltransferase, mainly involved in fatty acid degradation, were decreased by 0.2-, 0.3-, and 0.3-fold induced by MT, respectively. Meanwhile, enoyl-CoA hydratase, acyl-CoA synthetase, and short chain dehydrogenase, as the main enzymes for fatty acid synthesis, were increased by 0.4-, 0.3-, and 0.2-fold induced by MT treatment. However, delta 9 fatty acid desaturase, which introduces double bond in fatty acid at the ninth position, was decreased by 0.3-fold induced by MT treatment. This result indicates that MT can increase fatty acid synthesis and change fatty acid composition simultaneously. C22-sterol desaturase was the only DEG in sterol metabolism pathway, and its expression was decreased by 0.3-fold induced by MT treatment, indicating that MT could inhibit the sterol metabolism pathway which was a competing pathway with the astaxanthin synthesis to improve astaxanthin production. Amino acid transporter and ABC transporter, two of the most significant DEGs in substrate transportation in cell, were decreased by 1.2-fold and increased by 3.4-fold induced by MT treatment, respectively. In the anti-stress group, there were seven DEGs induced by MT-treatment. Except for the zinc-binding oxidoreductase with its expression level decreased by 0.3-fold, thioredoxin, heat shock protein 70, glutathione S-transferase, Svf1-like oxidative stress protein, Ish1-like stress-responsive protein, and manganese superoxide dismutase were increased by 0.4-, 0.4-, 0.7-, 0.3-, 0.3-, and 0.3-fold induced by MT treatment, respectively. This result indicates that MT activates the responses of cells to a series of stresses, such as temperature and oxygen stress. Rho GTPase-activating protein, serine threonine protein kinase, and srf-type transcription factor are important components in the mitogen-activated protein kinase (MAPK) signal pathway, and MT decreased their expressions by 0.4-, 0.4-, and 0.5-fold, respectively. In addition, iron/ascorbate oxidoreductase and calcium calmodulin-dependent protein kinase are essential components in the target of rapamycin (TOR) signal and calmodulin signal pathways, and their expressions are increased by 0.8- and 0.6-fold induced by MT treatment, respectively. This result implies that MT interacts with other signal pathways as a signaling molecule, activating the TOR and calmodulin signal pathways, while inhibiting the MAPK signal pathway. MT also changes the expression patterns of various transcription factors (TFs). For example, Zinc finger TF, pilin-like TF, and HMG-box TF were increased by 0.7-, 0.3-, and 0.2-fold, while the MEIS1 and heat shock TFs were decreased by 1.1- and 0.7-fold induced by MT treatment, respectively. To evaluate the accuracy of transcriptomic data, as shown in Table 1, the traditional RT-PCR analysis of all DEGs was performed, and the linear correlation analysis showed that the results from the FPKM and RT-PCR had significant correlation with an R value up to 0.9423 (Supplementary Figure S2). It is confirmed that the transcriptomic sequencing and analysis in this study have high quality and accuracy to lay the foundation for our future research.

**Table 1:** The differentially expressed genes (DEGs) which can explain astaxanthin improvement induced by melatonin (MT).

Category	Gene ID	Gene description	FPKM		log2(fc)	RT-PCR validation
			MT-treatment	MT-free		
Astaxanthin biosynthesis	4,765	Astaxanthin synthase	290.7 ± 33.8*	221.0 ± 24.1	0.4	0.5
	5,293	Geranyl pyrophosphate	111.0 ± 6.7*	92.7 ± 4.2	0.3	0.4
Fatty acid metabolism	11,707	Fatty acid-2 hydroxylase	1426.3 ± 17.5*	1627.2 ± 77.5	−0.2	−0.4
	6,377	Acyl-dehydrogenase	76.5 ± 6.6*	95.7 ± 13.9	−0.3	−0.4
	1,265	Acetyl-c-acyltransferase	185 ± 11.4*	231.1 ± 17.5	−0.3	−0.5
	519	Enoyl-CoA hydratase	31.2 ± 3.7*	24.0 ± 1.5	0.4	0.5
	4,739	Acyl-CoA synthetase	206.1 ± 6.0*	168.2 ± 20.9	0.3	0.2
	4,211	Delta 9 fatty acid desaturase	5358.9 ± 276.8*	6638.2 ± 210.0	−0.3	−0.5
	10,339	short chain dehydrogenase reductase	229.6 ± 15.5*	197.7 ± 22.5	0.2	0.1
Sterol metabolism	10,439	C22-sterol desaturase	290.1 ± 5.0*	362.7 ± 37.0	−0.3	−0.5
Substrate transportations	10,053	Amino acid transporters	0.1 ± 0.06**	0.3 ± 0.06	−1.2	−1.1
	1,699	ABC transporter	0.2 ± 0.1**	0.02 ± 0.03	3.4	3.4
Anti-stress	10,965	Thioredoxin	88.9 ± 7.7*	68.3 ± 3.4	0.4	0.2
	10,319	Heat shock protein 70	123.3 ± 2.6*	96.7 ± 16.9	0.4	0.1
	6,233	Glutathione S-transferase	252.5 ± 30.0*	157.2 ± 21.7	0.7	0.9
	11,591	Oxidative stress survival, Svf1-like	176.4 ± 7.0*	141.3 ± 31.7	0.3	0.5
	10,283	Zinc-binding oxidoreductase	62.4 ± 3.3*	77.4 ± 2.2	−0.3	−0.1
	8,715	Stress-responsive protein Ish1	750.4 ± 22.1*	617.6 ± 65.0	0.3	0.5
	9,027	Manganese superoxide dismutase	737.5 ± 51.7*	618.6 ± 10.2	0.3	0.1
Signal transduction	1,635	Iron/ascorbate family oxidoreductases	29.7 ± 2.7*	17.3 ± 2.0	0.8	0.6
	9,861	Calcium calmodulin-dependent protein kinase	20.4 ± 3.5*	13.1 ± 2.7	0.6	0.5
	9,743	Rho GTPase-activating protein	12.6 ± 0.9*	16.2 ± 1.6	−0.4	−0.1
	11,777	Serine threonine protein kinase	22.6 ± 2.0*	29.7 ± 5.8	−0.4	−0.9
TFs	609	Zinc finger	2.2 ± 0.2*	1.4 ± 0.3	0.7	0.3
	5,433	Pilin-like transcription factor	662.3 ± 43.2*	532.6 ± 66.2	0.3	0.3
	6,289	HMG-box transcription factor	22.0 ± 1.4*	18.6 ± 1.3	0.2	0.5
	6,823	Transcription factor MEIS1	0.5 ± 0.03**	1.0 ± 0.4	−1.1	−1.3
	5,667	Heat shock transcription factor	116.5 ± 12.0*	183.2 ± 73.4	−0.7	−0.9
	9,597	Srf-type transcription factor	12.4 ± 0.8*	17.1 ± 1.4	−0.5	−0.6

Data are given as means ± SD, *n* = 3. *p < 0.05; **p < 0.01.

### Enhancing astaxanthin biosynthesis through overexpression of a zinc finger TF

**Figure 5:**
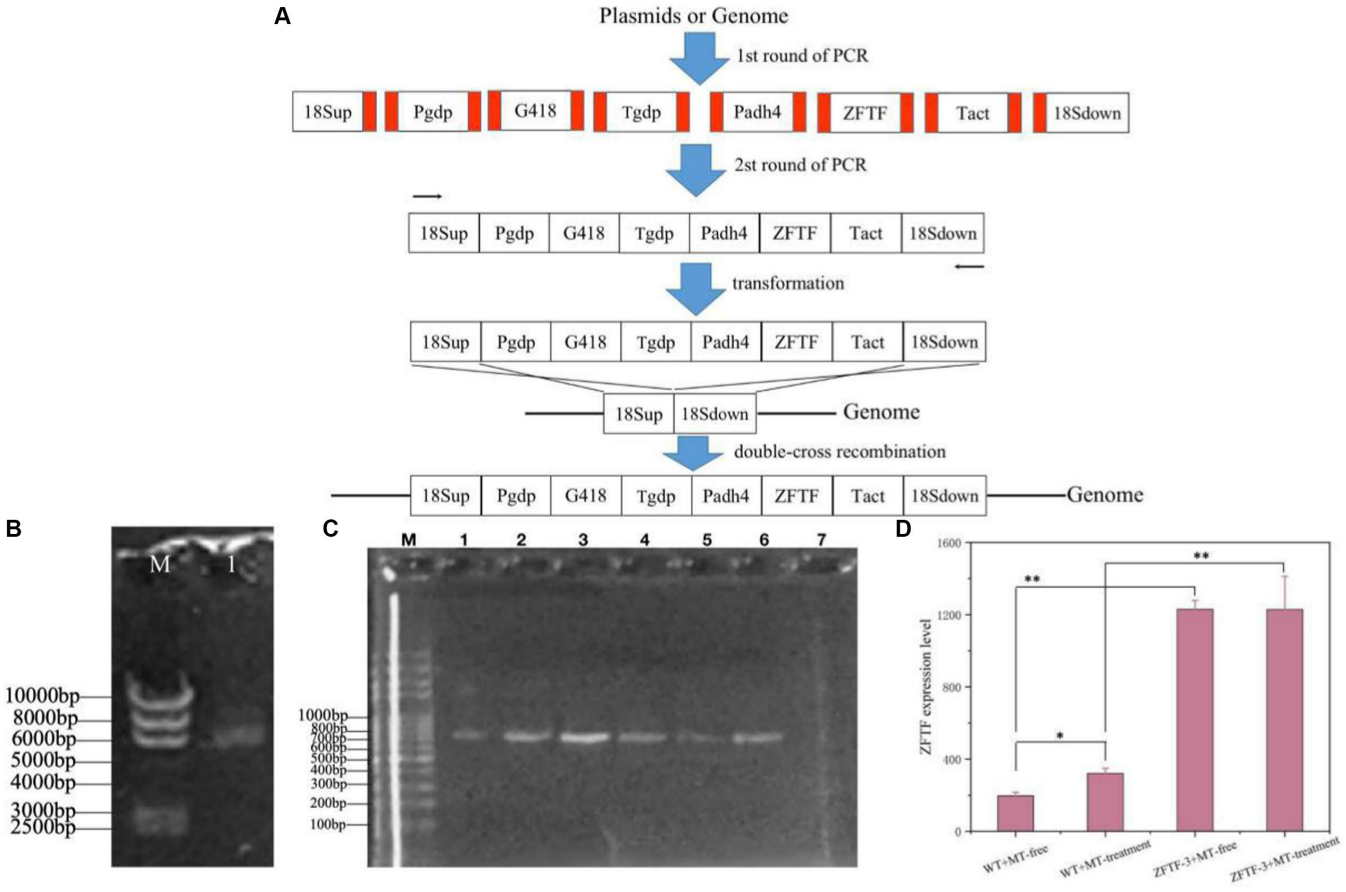
Construction of a ZFTF gene overexpression vector and its validations. **(A)** Schematic diagram of vector construction through 2-step overlap PCR. Red parts: homologous oligonucleotide with up-stream or down-stream DNA fragments. 18sup, Pgdp, G418, Tgdp, Padh4, ZFTF, Tact, and 18sdown: upstream of 18SrDNA, gdp gene promoter, G418-resistence gene, gdp gene terminator, adh4 gene promoter, zinc finger transcription factor gene, act gene terminator, and downstream of 18SrDNA. **(B)** Electrophoretic profile of the constructed ZFTF overexpression vector of 6,785 bp. (M) marker; 1, the vector containing 18sup, Pgdp, G418, Tgdp, Padh4, ZFTF, Tact, 18sdown fragments with a length of 6,785 bp. **(C)** Confirmations of ZFTF’s integrations into the 18SrDNA location of *P. rhodozyma* genome by PCR. M, marker; lane 1–6, transformants ZFTF-1 to ZFTF-6 carrying the ZFTF-overexpression vector; lane 8, the wild strain (WT) D, The *ZFTF* gene expressions of WT and transformant ZFTF-3 at the MT-free and MT treatment conditions analyzed by RT-PCR. Data are given as means ± SD, *n* = 3. The significances without annotation indicate the samples from MT treatment versus MT-free, and ZFTF overexpression versus none ZFTF. *p < 0.05; **p < 0.01.

**Figure 6:**
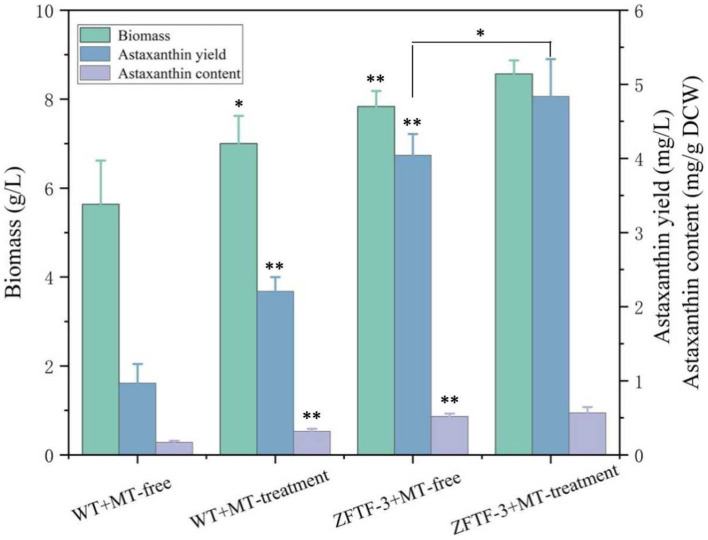
Biomass, astaxanthin content, and yield of *P. rhodozyma* transformant ZFTF-3 overexpressing a *ZFTF* gene under MT-free and MT treatment conditions. Data are given as means ± SD, *n* = 3. The significance without annotation indicate the samples from MT treatment versus MT-free and ZFTF overexpression versus none ZFTF. *p < 0.05; **p < 0.01.

TFs are the effectors of various signal transduction pathways and play important roles in regulations of numerous downstream genes at the global-cell level. Thus, a zinc finger TF (ZFTF), as a DEG with 0.7-fold increase induced by MT (Table 1), is selected to be overexpressed in *P. rhodozyma*. A rapid over-lap PCR method composed of 2-step PCR was used to construct a ZFTF overexpression cassette (Figure 5A). After the 2-step PCR performance, 6,785 bp of ZFTF overexpression cassette was constructed (Figure 5B). This DNA fragment was then transformed into the *P. rhodozyma* strain AS2.1557 (wide-type, WT), and six random transformants from ZFTF-1 to ZFTF-6 were selected for further analysis. A DNA fragment of approximately 800 bp covering the ZFTF, Tact, and 18sdown fragments could be amplified from the genomes of the six transformants while could not be amplified from the WT, indicating that the ZFTF overexpression cassette was inserted into the correct site of genome in *P. rhodozyma* (Figure 5C). A further biochemical analysis showed that biomass and astaxanthin production of the six transformants were higher than those of WT, indicating that the ZFTF expression level has positive effect on growth and astaxanthin synthesis in *P. rhodozyma* (data not shown). The transformant ZFTF-3 with the highest biomass and astaxanthin yield was selected for further analysis and validation. The RT-PCR analysis showed that expression levels of *ZFTF* gene in the ZFTF-3 strain under the MT-free condition were even up to 3.8-fold of the WT strain under the MT-treatment condition, indicating that the Padh4 is a strong promoter to efficiently drive the *ZFTF* gene expression with stronger induction effect under MT treatment. However, the *ZFTF* gene expression level in the transformant ZFTF-3 strain is not further increased under the MT-treatment condition, indicating that the Padh4 promoter is not inducible by MT treatment (Figure 5D). It is worth noting that biomass, astaxanthin content and yield of the transformant ZFTF-3 under MT-free condition could reach 4.8 g/L, 0.5 mg/g DCW and 4.0 mg/L, 39.1, 203.9 and 317.5% higher than those of WT strain under MT-free condition, even 11.9, 63.2 and 83.2% higher than those of WT strain under MT-treatment condition (Figure 6). This result shows that the ZFTF is a more efficient stimulator for biomass and astaxanthin synthesis in *P. rhodozyma*. In other words, the ZFTF gene overexpression is a more essential factor contributing to the improvement of astaxanthin synthesis in *P. rhodozyma*. Under the MT treatment, although astaxanthin content of the transformant ZFTF-3 was not further significantly increased compared with its counterpart under the MT-free condition, a further 9.4% increase in biomass resulted in a further 19.7% increase in astaxanthin yield. Summarily, the biomass, astaxanthin content, and yield in the transformant ZFTF-3 under MT treatment condition reached up to 8.6 g/L, 0.6 mg/g DCW, and 4.8 mg/L, 52.1, 233.3, and 399.7% higher than those in WT strain under MT-free condition. Thus, in this study, we present an efficient synergistic strategy of MT treatment and zinc finger transcription factor (*ZFTF*) gene overexpression in *P. rhodozyma* for astaxanthin production.

## Discussion

In addition to higher plants, melatonin (MT) has also been found to act on some species closely related to the higher plants. As a lower plant, the microalgae *H. pluvialis* could also respond to 10 μM (~2.5 mg/L) exogenous MT to increase its astaxanthin production by 2.36-fold, with no positive or even negative effects on its biomass under abiotic stresses, such as limited nitrogen and high light. Further analysis showed that MT stimulated the second messenger nitric oxide (NO) and salicylic acid (SA) to upregulate the expression of astaxanthin biosynthesis genes (Ding et al., 2018a). Similarly, as a producer of astaxanthin, *P. rhodozyma* is originated from plant materials and has evolved to adapt the metabolites from its symbiotic plant (Bellora et al., 2016). Thus, various plant extracts or phytohormones have been proven to have positive effects on biomass and astaxanthin synthesis as a potential strategy to breakthrough astaxanthin bottleneck in *P. rhodozyma* (Kim et al., 2007; Stachowiak, 2012; Kothari et al., 2019; Pan et al., 2020; Liu et al., 2022). Although Yang et al. analyzed the effects of MT on biomass and astaxanthin content in *P. rhodozyma*, the mechanism of MT regulating astaxanthin synthesis in this yeast at global level was still unclear (Yang et al., 2023). In this study, a concentration below 1 mg/L MT could increase the biomass, astaxanthin content, and yield in *P. rhodozyma* by 21.9, 93.9, and 139.1%, respectively, with the same order of magnitude and even lower dose than the previous reports (Ding et al., 2018a; Yang et al., 2023). Moreover, unlike no effect or even negative effect of MT on biomass in *P. rhodozyma* or *H. pluvialis*, the biomass in *P. rhodozyma* strain AS2.1557 is induced by MT treatment. These results imply that *P. rhodozyma* strain AS2.1557 is more sensitive to MT than the *P. rhodozyma* strain JMU-MVP14 and *H. pluvialis*, and the effects of MT on biomass and astaxanthin synthesis in *P. rhodozyma* are strain-specific. Thus, the mechanism of MT improving biomass and astaxanthin synthesis in *P. rhodozyma* should be further analyzed.

A further RNA-seq-based transcriptomic analysis showed that MT could change transcriptomic profile at the global-cell level, and the differentially expressed genes (DEGs) induced by MT were also identified. Surprisingly, only astaxanthin synthase and geranyl pyrophosphate synthase in astaxanthin synthesis pathway were identified as the upregulated DEGs with the other genes not significantly changed, indicating that MT regulate the global-cell metabolic network which was not only the single astaxanthin synthesis pathway to improve astaxanthin production. As shown in Table 1, there are seven DEGs in fatty acid metabolism category. Among these, four DEGs involved in fatty acid degradation were inhibited by MT, while three DEGs involved in fatty acid synthesis were induced by MT. Although fatty acids are the competing metabolites and their synthesis share the same precursor with astaxanthin synthesis, induction of the fatty acid synthesis and inhibition of the fatty acid degradation by MT implied that MT treatment improves the fatty acid and astaxanthin synthesis simultaneously. Our result was consistent with the result reported by Liu et al. that gibberellic acid (GA) could improve the fatty acid and astaxanthin synthesis simultaneously (Liu et al., 2022). The result can be explained by the fact that astaxanthin is dissolved in lipid droplets to keep stable; hence, fatty acid synthesis contributes to the accumulation of astaxanthin (Zhao et al., 2020). On the contrary, fatty acid is a competitive metabolite for astaxanthin, and a reduction in this pathway can increase astaxanthin production (Miao et al., 2011). The decrease in carbon flux from fatty acid synthesis may also lead to an increase in the other carotenoid pathways to increase astaxanthin production (Zhang et al., 2020b). These results show that the balance between fatty acid and astaxanthin synthesis is an important factor for regulating astaxanthin synthesis in *P. rhodozyma*. In this study, inhibition of the sterol synthesis pathway (C22-sterol desaturase as a downregulated DEG) by MT treatment was similar with the result reported by Liu et al. that the *egr7* gene (encoding lanosterol synthase in the steroid biosynthesis pathway) was significantly downregulated by GA treatment to increase astaxanthin synthesis (Liu et al., 2022). These results indicate that the sterol pathway is a negative regulation target for improving astaxanthin synthesis in *P. rhodozyma*.

ABC transporter is responsible for accumulation and transmembrane transport of secondary metabolites and the most upregulated DEGs, as shown in Table 1 (3.4-fold) (Theodoulou and Kerr, 2015). Carotenoids, including astaxanthin, are not automatically released from the cells, thus increased accumulation of astaxanthin in the cells can induce toxicity (Lee et al., 2016). Induction of ABC transporter by MT treatment can relieve the toxicity issue caused by astaxanthin accumulation as a cellular anti-stress response to maintain or even improve cellular growth. Overexpression of ABC transporters can also promote the secretion of carotene in *Saccharomyces cerevisiae* (Bu et al., 2020). Thus, metabolite-transportation engineering in *P. rhodozyma* will be an ideal strategy for improving astaxanthin yield in the future study. On the contrary, amino acid transporter is one of the most downregulated DEGs induced by MT treatment (Table 1). After acylation by carboxyl groups of lysine, water-dispersible solubility and/or dispersibility of astaxanthin are significantly improved, thereby maintaining its function and stability. Therefore, as a conjugate of astaxanthin, amino acids are of great significance for maintaining its activity and homeostasis (Jackson et al., 2004). The amino acid transporter (AAT) is mainly responsible for the transmembrane transport of amino acids and decrease in its expression levels may cause more amino acids to accumulate in the cell and conjugate with more astaxanthin, maintaining cellular homeostasis (Kandasamy et al., 2018).

**Figure 7:**
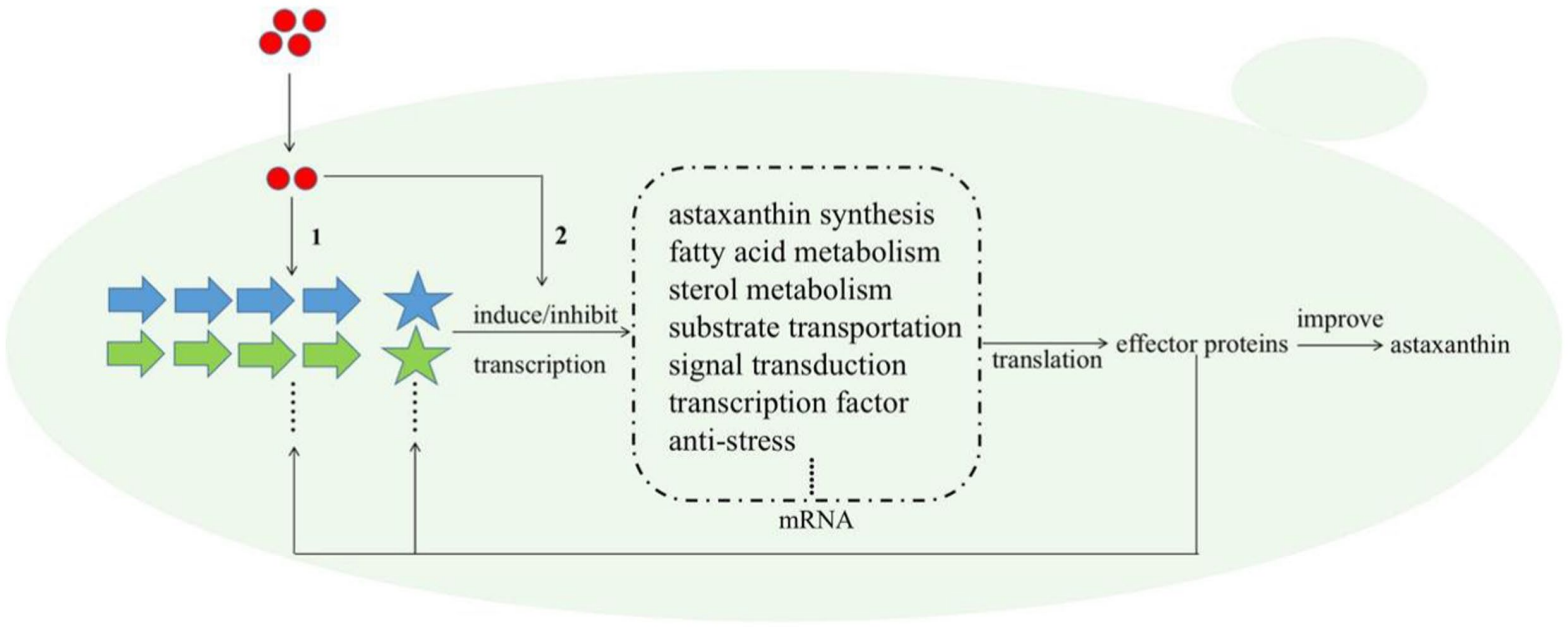
Schematic diagram for the molecular mechanism of MT improving astaxanthin synthesis in *P. rhodozyma* at the global-cell level. Red circle, melatonin (MT); large arrows with different colors, various signal pathways; pentagrams, transcription factors (TFs) of different signal pathways with the same color as their related signal pathways; dashed box, the target genes or DEGs induced by MT. The mechanism of MT regulating astaxanthin biosynthesis is that (1) MT interact with the components of related signal pathways to alter signal transduction, or (2) MT as a signal molecule directly regulate the transcriptional levels of the target genes. The changed signal transduction transmitted to transcription factors (TFs), and TFs regulate the expressions of target genes at transcriptional level, leading to the quantity changes in related effector proteins. A part of effector proteins are components or TFs of related signal pathways, which reversely affect the signal pathways and regulate the expressions of target genes, while other parts of effector proteins regulate astaxanthin synthesis.

Melatonin (MT) is an important messenger in higher plants and plays an essential role in their resistances to abiotic and biological stresses. In this study, we found that MT could stimulate up to six anti-stress-related genes (Table 1); meanwhile, astaxanthin and even the cell growth under the stress condition were all increased by MT treatment (Figure 6). Astaxanthin is a well-known super antioxidant, and the six DEGs induced by MT encode proteins that are resistant to various stresses, such as oxygen, temperature, and nutrition deficiency. These results indicate that MT fully activate the anti-stress response of *P. rhodozyma* and even improve its growth under the stress condition, which is beneficial for astaxanthin synthesis. As a messenger, the effects and regulations of MT on signaling pathways are the focus of this study. It is confirmed that MT can activate the TOR and calmodulin signal pathways and inhibit the MAPK signal pathway by identifying the DEGs of signal pathways and transcription factors (TFs) induced by MT (Table 1). In *H. pluvialis*, MT was found to stimulate the cAMP signaling pathway and astaxanthin accumulation to stimulate the NO-dependent MAPK signaling pathway, confirming that MAPK is a target NO action in physiological processes (Ding et al., 2018b). Thus, we propose the mechanism of MT regulating astaxanthin synthesis in *P. rhodozyma*; MT enters into the cells as a messenger and interact with the components of other signaling pathways to affect their signal transduction. Alternatively, MT directly regulates gene expressions of the components in the other signaling pathways to affect their signal transduction. The affected signals are then transmitted to transcription factors (TFs), and TFs regulate expression profiles of the downstream target genes. The downstream regulated genes are the response mechanism of yeast cells to MT treatment (Figure 7).

Transcription factors (TFs) are regulators to alter complex microbial metabolic pathways and can simultaneously activate or repress multiple genes in specific pathways, enforcing the target metabolite synthesis and improving the stress tolerance. Transcription factor engineering (TFE) is proven to be an ideal strategy for microbial synthesis and metabolism modification (Deng et al., 2022). Although the TFE has been widely applied in genetic engineering modification of yeast and microalgae, various TFs regulating microbial astaxanthin synthesis have been continuously being discovered; the genetic modification of astaxanthin biosynthesis based on TFE technology has not been reported (Grama et al., 2022). The potential of TFE to increase astaxanthin productivity in *H. pluvialis* has been recognized and proposed (Kayani et al., 2023). In this study, a biochemical and genetic strategy based on hormone treatment and TFE is applied in the astaxanthin-producing yeast *P. rhodozyma* for the first time and simultaneously increased its cell growth and astaxanthin synthesis, confirming that our strategy is efficient and lay the foundation for microbial astaxanthin production.

## Conclusion

Melatonin (MT) with a concentration of 1 mg/L can simultaneously improve cell growth and astaxanthin synthesis in *P. rhodozyma*. Moreover, MT can perturbate the global transcriptomic profile of *P. rhodozyma* and the differentially expressed genes (DEGs). These DEGs induced by MT are involved in astaxanthin synthesis, metabolite metabolism, anti-stress, signal transduction, and transcription factors. Overexpression of a zinc finger transcription factor (ZFTF), which is significantly induced by MT and serves as one of the most upregulated DEGs, can further increase biomass and astaxanthin synthesis in *P. rhodozyma*, resulting in a higher astaxanthin yield.

## Data availability statement

The datasets presented in this study can be found in online repositories. The names of the repository/repositories and accession number(s) can be found in the article/Supplementary material. The RNA-seq raw data have been uploaded to the Sequence Read Archive (SRA) database, and the reference number is PRJNA1076472 (SRR27966608–27966613).

## Author contributions

JJ: Data curation, Formal analysis, Investigation, Methodology, Software, Validation, Writing – original draft, Writing – review & editing. ZC: Formal analysis, Investigation, Methodology, Software, Validation, Writing – original draft. QL: Conceptualization, Data curation, Formal analysis, Methodology, Software, Writing – review & editing. FL: Writing – review & editing. SL: Data curation, Formal analysis, Investigation, Methodology, Writing – review & editing. GB: Conceptualization, Funding acquisition, Project administration, Resources, Supervision, Writing – original draft, Writing – review & editing.

## Footnotes

^1^ https://github.com/OpenGene/fastp

^2^ https://ccb.jhu.edu/software/hisat2

^3^ https://bioconductor.org/packages/release/bioc/html/edgeR.html

^4^Kyoto Encyclopedia of Genes and Genomes, http://en.wikipedia.org/wiki/KEGG

## References

[ref1] Adrio J. L. Veiga M. (1995). Transformation of the astaxanthin-producing yeast *Phaffia rhodozyma*. Biotechnol. Tech. 9, 509–512. doi: 10.1007/BF00159567 7586031

[ref2] Basiony M. Ouyang L. Wang D. Yu J. Zhou L. Zhu M.. (2022). Optimization of microbial cell factories for astaxanthin production: biosynthesis and regulations, engineering strategies and fermentation optimization strategies. Synth. Syst. Biotechnol. 7, 689–704. doi: 10.1016/j.synbio.2022.01.002, PMID: 35261927 PMC8866108

[ref3] Bellora N. Moliné M. David-Palma M. Coelho M. A. Hittinger C. T. Sampaio J. P.. (2016). Comparative genomics provides new insights into the diversity, physiology, and sexuality of the only industrially exploited tremellomycete: *Phaffia rhodozyma*. BMC Genomics 17:901. doi: 10.1186/s12864-016-3244-7, PMID: 27829365 PMC5103461

[ref4] Breitenbach J. Pollmann H. Sandmann G. (2019). Genetic modification of the carotenoid pathway in the red yeast *Xanthophyllomyces dendrorhous*: engineering of a high-yield zeaxanthin strain. J. Biotechnol. 289, 112–117. doi: 10.1016/j.jbiotec.2018.11.019, PMID: 30496776

[ref5] Bu X. Lin J.-Y. Cheng J. Yang D. Duan C.-Q. Koffas M.. (2020). Engineering endogenous ABC transporter with improving ATP supply and membrane flexibility enhances the secretion of β-carotene in *Saccharomyces cerevisiae*. Biotechnol. Biofuels 13:168. doi: 10.1186/s13068-020-01809-6, PMID: 33062054 PMC7548044

[ref6] Debnath B. Islam W. Li M. Sun Y. Lu X. Mitra S.. (2019). Melatonin mediates enhancement of stress tolerance in plants. Int. J. Mol. Sci. 20. doi: 10.3390/ijms20051040, PMID: 30818835 PMC6429401

[ref7] Deng C. Wu Y. Lv X. Li J. Liu Y. Du G.. (2022). Refactoring transcription factors for metabolic engineering. Biotechnol. Adv. 57:107935. doi: 10.1016/j.biotechadv.2022.107935, PMID: 35271945

[ref8] Ding W. Zhao P. Peng J. Zhao Y. Xu J.-W. Li T.. (2018a). Melatonin enhances astaxanthin accumulation in the green microalga *Haematococcus pluvialis* by mechanisms possibly related to abiotic stress tolerance. Algal Res. 33, 256–265. doi: 10.1016/j.algal.2018.05.021

[ref9] Ding W. Zhao Y. Xu J.-W. Zhao P. Li T. Ma H.. (2018b). Melatonin: a multifunctional molecule that triggers defense responses against high light and nitrogen starvation stress in *Haematococcus pluvialis*. J. Agric. Food Chem. 66, 7701–7711. doi: 10.1021/acs.jafc.8b02178, PMID: 29975059

[ref10] Gómez M. Campusano S. Gutiérrez M. S. Sepúlveda D. Barahona S. Baeza M.. (2020). Sterol regulatory element-binding protein Sre1 regulates carotenogenesis in the red yeast *Xanthophyllomyces dendrorhous*. J. Lipid Res. 61, 1658–1674. doi: 10.1194/jlr.RA120000975, PMID: 32933952 PMC7707178

[ref11] Grama S. B. Liu Z. Li J. (2022). Emerging trends in genetic engineering of microalgae for commercial applications. Mar. Drugs 20:285. doi: 10.3390/md20050285, PMID: 35621936 PMC9143385

[ref12] Hara K. Y. Morita T. Endo Y. Mochizuki M. Araki M. Kondo A. (2014). Evaluation and screening of efficient promoters to improve astaxanthin production in *Xanthophyllomyces dendrorhous*. Appl. Microbiol. Biotechnol. 98, 6787–6793. doi: 10.1007/s00253-014-5727-2, PMID: 24737060

[ref13] Hussein G. Sankawa U. Goto H. Matsumoto K. Watanabe H. (2006). Astaxanthin, a carotenoid with potential in human health and nutrition. J. Nat. Prod. 69, 443–449. doi: 10.1021/np050354+, PMID: 16562856

[ref14] Igreja W. S. Maia F. D. Lopes A. S. Chiste R. C. (2021). Biotechnological production of carotenoids using low cost-substrates is influenced by cultivation parameters: a review. Int. J. Mol. Sci. 22. doi: 10.3390/ijms22168819, PMID: 34445525 PMC8396175

[ref15] Jackson H. L. Cardounel A. J. Zweier J. L. Lockwood S. F. (2004). Synthesis, characterization, and direct aqueous superoxide anion scavenging of a highly water-dispersible astaxanthin-amino acid conjugate. Bioorganic Med. Chem. Lett. 14, 3985–3991. doi: 10.1016/j.bmcl.2004.05.038, PMID: 15225712

[ref16] Kandasamy P. Gyimesi G. Kanai Y. Hediger M. A. (2018). Amino acid transporters revisited: new views in health and disease. Trends Biochem. Sci. 43, 752–789. doi: 10.1016/j.tibs.2018.05.003, PMID: 30177408

[ref17] Kayani S. I. Rahman S. U. Shen Q. Cui Y. Liu W. Hu X.. (2023). Molecular approaches to enhance astaxanthin biosynthesis; future outlook: engineering of transcription factors in *Haematococcus pluvialis*. Crit. Rev. Biotechnol. 1-16, 1–16. doi: 10.1080/07388551.2023.2208284, PMID: 37380353

[ref18] Kim S. K. Lee J. H. Lee C. H. Yoon Y. C. (2007). Increased carotenoid production in *Xanthophyllomyces dendrorhous* G276 using plant extracts. J. Microbiol. 45, 128–132. PMID: 17483797

[ref19] Kizer L. Pitera D. J. Pfleger B. F. Keasling J. D. (2008). Application of functional genomics to pathway optimization for increased isoprenoid production. Appl. Environ. Microbiol. 74, 3229–3241. doi: 10.1128/AEM.02750-07, PMID: 18344344 PMC2394933

[ref20] Kothari D. Lee J.-H. Chon J.-W. Seo K.-H. Kim S.-K. (2019). Improved astaxanthin production by *Xanthophyllomyces dendrorhous* SK984 with oak leaf extract and inorganic phosphate supplementation. Food Sci. Biotechnol. 28, 1171–1176. doi: 10.1007/s10068-019-00604-w, PMID: 31275717 PMC6595044

[ref21] Lee J. J. L. Chen L. Cao B. Chen W. N. (2016). Engineering *Rhodosporidium toruloides* with a membrane transporter facilitates production and separation of carotenoids and lipids in a bi-phasic culture. Appl. Microbiol. Biotechnol. 100, 869–877. doi: 10.1007/s00253-015-7102-3, PMID: 26526454

[ref22] Li B. Dewey C. N. (2011). RSEM: accurate transcript quantification from RNA-Seq data with or without a reference genome. BMC Bioinform. 12:323. doi: 10.1186/1471-2105-12-323, PMID: 21816040 PMC3163565

[ref23] Li W. Luna-Flores C. H. Anangi R. Zhou R. Tan X. Jessen M.. (2023). Oxidative stress induced by plasma-activated water stimulates astaxanthin production in *Phaffia rhodozyma*. Bioresour. Technol. 369:128370. doi: 10.1016/j.biortech.2022.128370, PMID: 36423765

[ref24] Li D. Zhao Y. Ding W. Zhao P. Xu J. W. Li T.. (2017). A strategy for promoting lipid production in green microalgae *Monoraphidium* sp. QLY-1 by combined melatonin and photoinduction. Bioresour. Technol. 235, 104–112. doi: 10.1016/j.biortech.2017.03.114, PMID: 28365337

[ref25] Liu Y. S. Wu J. Y. (2006). Use of n-hexadecane as an oxygen vector to improve *Phaffia rhodozyma* growth and carotenoid production in shake-flask cultures. J. Appl. Microbiol. 101, 1033–1038. doi: 10.1111/j.1365-2672.2006.03009.x, PMID: 17040227

[ref26] Liu Y. S. Wu J. Y. (2007). Perfusion culture process plus H2O2 stimulation for efficient astaxanthin production by *Xanthophyllomyces dendrorhous*. Biotechnol. Bioeng. 97, 568–573. doi: 10.1002/bit.21256, PMID: 17149775

[ref27] Liu S. Yi H. Zhan H. Wang L. Wang J. Li Y.. (2022). Gibberellic acid-induced fatty acid metabolism and ABC transporters promote astaxanthin production in *Phaffia rhodozyma*. J. Appl. Microbiol. 132, 390–400. doi: 10.1111/jam.15187, PMID: 34161638

[ref28] Livak K. J. Schmittgen T. D. (2001). Analysis of relative gene expression data using real-time quantitative PCR and the 2−ΔΔCT method. Methods 25, 402–408. doi: 10.1006/meth.2001.1262 11846609

[ref29] Mao X. Cai T. Olyarchuk J. G. Wei L. (2005). Automated genome annotation and pathway identification using the KEGG Orthology (KO) as a controlled vocabulary. Bioinformatics 21, 3787–3793. doi: 10.1093/bioinformatics/bti430, PMID: 15817693

[ref30] Miao L. Chi S. Tang Y. Su Z. Yin T. Guan G.. (2011). Astaxanthin biosynthesis is enhanced by high carotenogenic gene expression and decrease of fatty acids and ergosterol in a *Phaffia rhodozyma* mutant strain. FEMS Yeast Res. 11, 192–201. doi: 10.1111/j.1567-1364.2010.00705.x, PMID: 21155970

[ref31] Naguib Y. M. A. (2000). Antioxidant activities of Astaxanthin and related carotenoids. J. Agric. Food Chem. 48, 1150–1154. doi: 10.1021/jf991106k 10775364

[ref32] Niklitschek M. Alcaíno J. Barahona S. Sepúlveda D. Lozano C. Carmona M.. (2008). Genomic organization of the structural genes controlling the astaxanthin biosynthesis pathway of *Xanthophyllomyces dendrorhous*. Biol. Res. 41, 93–108. doi: 10.4067/s0716-97602008000100011, PMID: 18769767

[ref33] Nutakor C. Kanwugu O. N. Kovaleva E. G. Glukhareva T. V. (2022). Enhancing astaxanthin yield in *Phaffia rhodozyma*: current trends and potential of phytohormones. Appl. Microbiol. Biotechnol. 106, 3531–3538. doi: 10.1007/s00253-022-11972-5, PMID: 35579685

[ref34] Pan X. Wang B. Duan R. Jia J. Li J. Xiong W.. (2020). Enhancing astaxanthin accumulation in *Xanthophyllomyces dendrorhous* by a phytohormone: metabolomic and gene expression profiles. Microb. Biotechnol. 13, 1446–1460. doi: 10.1111/1751-7915.13567, PMID: 32426951 PMC7415379

[ref35] Poeggeler B. Thuermann S. Dose A. Schoenke M. Burkhardt S. Hardeland R. (2002). Melatonin's unique radical scavenging properties - roles of its functional substituents as revealed by a comparison with its structural analogs. J. Pineal Res. 33, 20–30. doi: 10.1034/j.1600-079x.2002.01873.x, PMID: 12121482

[ref36] Ramirez J. Nunez M. Valdivia R. (2000). Increased astaxanthin production by a *Phaffia rhodozyma* mutant grown on date juice from *Yucca fillifera*. J. Ind. Microbiol. Biotechnol. 24, 187–190. doi: 10.1038/sj.jim.2900792

[ref37] Shi H. Tan D. X. Reiter R. J. Ye T. Yang F. Chan Z. (2015). Melatonin induces class A1 heat-shock factors (HSFA1s) and their possible involvement of thermotolerance in Arabidopsis. J. Pineal Res. 58, 335–342. doi: 10.1111/jpi.12219, PMID: 25711624

[ref38] Stachowiak B. (2012). Astaxanthin synthesis by yeast *Xanthophyllomyces dendrorhous* and its mutants on media based on plant extracts. Indian J. Microbiol. 52, 654–659. doi: 10.1007/s12088-012-0306-7, PMID: 24293726 PMC3516669

[ref39] Theodoulou F. L. Kerr I. D. (2015). ABC transporter research: going strong 40 years on. Biochem. Soc. Trans. 43, 1033–1040. doi: 10.1042/bst20150139, PMID: 26517919 PMC4652935

[ref40] Wozniak A. Lozano C. Barahona S. Niklitschek M. Marcoleta A. Alcaíno J.. (2011). Differential carotenoid production and gene expression in *Xanthophyllomyces dendrorhous* grown in a nonfermentable carbon source. FEMS Yeast Res. 11, 252–262. doi: 10.1111/j.1567-1364.2010.00711.x, PMID: 21205159

[ref41] Xie S.-R. Li Y. Chen H.-H. Liang M.-H. Jiang J.-G. (2022). A strategy to promote carotenoids production in *Dunaliella bardawil* by melatonin combined with photoinduction. Enzym. Microb. Technol. 161:110115. doi: 10.1016/j.enzmictec.2022.110115, PMID: 36030697

[ref42] Yang H. Yang L. Du X. He N. Jiang Z. Zhu Y.. (2023). Metabolomics of astaxanthin biosynthesis and corresponding regulation strategies in *Phaffia rhodozyma*. Yeast 40, 254–264. doi: 10.1002/yea.3854, PMID: 37132227

[ref43] Yu X.-J. Chen H. Huang C.-Y. Zhu X.-Y. Wang Z.-P. Wang D.-S.. (2019). Transcriptomic mechanism of the Phytohormone 6-Benzylaminopurine (6-BAP) stimulating lipid and DHA synthesis in *Aurantiochytrium* sp. J. Agric. Food Chem. 67, 5560–5570. doi: 10.1021/acs.jafc.8b07117, PMID: 30901205

[ref44] Yu X.-J. Wang Z.-P. Liang M.-J. Wang Z. Liu X.-Y. Hu L.. (2020). One-step utilization of inulin for docosahexaenoic acid (DHA) production by recombinant *Aurantiochytrium* sp. carrying *Kluyveromyces marxianus* inulinase. Bioprocess Biosyst. Eng. 43, 1801–1811. doi: 10.1007/s00449-020-02371-z, PMID: 32405771

[ref45] Zhang J. Li Q. Liu J. Lu Y. Wang Y. Wang Y. (2020a). Astaxanthin overproduction and proteomic analysis of *Phaffia rhodozyma* under the oxidative stress induced by TiO2. Bioresour. Technol. 311:123525. doi: 10.1016/j.biortech.2020.123525, PMID: 32447228

[ref46] Zhang Y. Ye Y. Ding W. Mao X. Li Y. Gerken H.. (2020b). Astaxanthin is Ketolated from zeaxanthin independent of fatty acid synthesis in *Chromochloris zofingiensis*. Plant Physiol. 183, 883–897. doi: 10.1104/pp.20.00325, PMID: 32385091 PMC7333715

[ref47] Zhao Y. Hou Y. Chai W. Liu Z. Wang X. He C.. (2020). Transcriptome analysis of *Haematococcus pluvialis* of multiple defensive systems against nitrogen starvation. Enzym. Microb. Technol. 134:109487. doi: 10.1016/j.enzmictec.2019.109487, PMID: 32044034

