# Supplementary material for: The enhancement of astaxanthin production in Phaffia rhodozyma through a synergistic melatonin treatment and zinc finger transcription factor gene overexpression
Source: Front Microbiol. 2024 Apr 11;15:1367084. doi: 10.3389/fmicb.2024.1367084 (PMC11043562; doi:10.3389/fmicb.2024.1367084)
Supplement: Supplementary file 1 [file Data_Sheet_1.docx]

Table S1 Quality control of the transcriptomic data in this study

| Sample | Raw Data | | Valid Data | | Valid Ratio(reads) | Q20% | Q30% | GC content% |
| --- | --- | --- | --- | --- | --- | --- | --- | --- |
|  | Read | Base | Read | Base |  |  |  |  |
| C1 | 43180242 | 6.48G | 42175042 | 6.33G | 97.67 | 99.96 | 97.84 | 51 |
| C2 | 39508668 | 5.93G | 38386514 | 5.76G | 97.16 | 99.95 | 97.74 | 51.5 |
| C3 | 36588658 | 5.49G | 35187812 | 5.28G | 96.17 | 99.96 | 97.89 | 52 |
| M1 | 40476680 | 6.07G | 39454000 | 5.92G | 97.47 | 99.95 | 97.87 | 52 |
| M2 | 46346934 | 6.95G | 44118686 | 6.62G | 95.19 | 99.96 | 97.91 | 52 |
| M3 | 43745514 | 6.56G | 42538470 | 6.38G | 97.24 | 99.95 | 97.83 | 52 |

Table S2 The primers used for RT-PCR in this study

| Gene | Primers |
| --- | --- |
| astaxanthin synthase | F-TCTTGCTCACAGGTGCTTTAGGC |
|  | R-TGCAAACGCATCTGGTACCCC |
| geranyl pyrophosphate | F-AGTTCGAGGCTGTCTTCCCG |
|  | R-TATAGACCTTACCCAGGAAGCTGTGG |
| fatty acid-2 hydroxylase | F-AATTACTAAGAAGGCCAGAATCTATGCGG |
|  | R-TGTCTGTGAACCTCTTGATGAAAACGTTCC |
| acyl-dehydrogenase | F-CCCAAGCAGAGCTTCAGGAGT |
|  | R-CCTTTGGGAATTTTTTTCTGGGCTTG |
| acetyl-c-acyltransferase | F-AAGTCCAAGATCTTGCAAAAGAACGC |
|  | R-AAGACCCATACCAGACCCAATGCA |
| Enoyl-CoA hydratase | F-TGCTTTGTTCCCGACAGGGG |
|  | R-AAGTTAGGAGCTCTTTTCTCAACGAACG |
| Acyl-CoA synthetase | F-GCCTCTCTCCTTAGAGGAAGAGTAC |
|  | R-GTAGAGAGCCCTGGTTGAACCAT |
| Delta 9 fatty acid desaturase | F-AAATGAAACAGCGACCTCGGTC |
|  | R-GAATCCCATGCCGATGATCAGGT |
| short chain dehydrogenase reductase | F-AGATTCTTGCAATTCGATCTTTCCACC |
|  | R-ATGGTCTTTCATACCTCCGGTCATATC |
| C22-sterol desaturase | F-TCAACACGTACCAGCGCCTTC |
|  | R-CATGTAAGGCTTGGGCTCGGT |
| Amino acid transporters | F-CTGGACGACGATGAGTATGACGT |
|  | R-CTCCGGGATCTTTCCAGTAGCG |
| ABC transporter | F-CAGGTACGCGCTACAAGCAG |
|  | R-GTGGCCTCTCTTGATTCCGAG |
| Thioredoxin | F-AGTCTTTTGAGGAGTTCAAGAACATCATCA |
|  | R-TTCCTGGACAGCAGGCTCGG |
| heat shock protein 70 | F-AATACGGAACTGTTATCGGTATTGATTTGGG |
|  | R-GTGAGCAGAGACCTTCATGATACCG |
| Glutathione S-transferase | F-CCATATTTCTGGAAGAACTCAAAGCGG |
|  | R-CTTTCATCGTTGGCTCTCTCTCCAG |
| Oxidative stress survival, Svf1-like | F-AACTTCAAGTCTCCTCCTACTTCCG |
|  | R-AGGGAATCTCGGCCAACAC |
| Zinc-binding oxidoreductase | F-ACCAGCTCGCTTCCCAAGA |
|  | R-AGTTGATCGTCTCCAGTCGGCT |
| Stress-responsive protein Ish1 | F-TCCCTCTCGCCACTGTGGTAT |
|  | R-GACACCGGCAAGAGCATCAAGAATAC |
| manganese superoxide dismutase | F-TTACGCTTACGATGCCTTGGAGC |
|  | R-CAGCATCAAACCTTCGCTGAGCC |
| Iron/ascorbate family oxidoreductases | F-ATCATATTCCCATCATCGATCTTGCG |
|  | R-ATTCACCCGCGAGGATGGGT |
| calcium calmodulin-dependent protein kinase | F-AAACTGGAACGACGGCCGA |
|  | R-AACTCCGACTGAGCGCCATTA |
| Rho GTPase-activating protein | F-AAGGAAGTGCCGCTCACTCG |
|  | R-TAAATGAAAAGTAAAGTCCAAGACTAGGAAATCGG |
| serine threonine protein kinase | F-ACACCCTTCGATGTCCTCCGCA |
|  | R-TAAGGACGGAAAGCAAGTCTGGATG |
| Zinc finger | F-TCGCGGAGGCTAAGTGGCTGA |
|  | R-TTCCTTTTCGACATACTCGGGCAGT |
| pilin-like transcription factor | F-CTTGCCCGGCTCACTCAAGC |
|  | R-TCCACAAGCGGTGCAAGTGATCT |
| HMG-box transcription factor | F-AGTCCAACAGCGCCTCCAAT |
|  | R-CGGTGGTCGGGATAGGTATTGGG |
| Transcription factor MEIS1 | F-ATCTCTCTGCCCGCTTAGACTCT |
|  | R-ATTTACCGAGAACCTGGTAGATTGTAATG |
| heat shock transcription factor | F-ACAGGGCATCTTGCTCTGTCCT |
|  | R-AACTAGCGACATTTGAGTGTCTGAAGA |
| srf-type transcription factor | F-AATCTTACGGTGGCATCAATACAACATG |
|  | R-TGAGACCCGAAGGGTAATTTGTTTGTG |

Table S3 The primers used for construction of the zinc finger transcription factor (ZFTF) gene overexpression vector

| Primers | Sequence |
| --- | --- |
| 18sup-F | AGTCATATGCTTGTCTCAAAGATTAAGCCA |
| 18sup-R | TCTCGGGTTCCCTCGACTTGTCATTACGGCGATCCTAGAAACCAA |
| Pgpd-F | TTGGTTTCTAGGATCGCCGTAATGACAAGTCGAGGGAACCCGAGA |
| Pgpd-R | AGCAAGACGTTTCCCGTTCATGATGGTAAGAGTGTTAGAGAAGTAGTGGTGG |
| G418-F | CCACCACTACTTCTCTAACACTCTTACCATCATGAACGGGAAACGTCTTGCT |
| G418-R | AGAGGGTTTGGAGAGAACCGTTTATAACCAATTCTGATTAGAAAAACTCATCGAGCA |
| Tgpd-F | TGCTCGATGAGTTTTTCTAATCAGAATTGGTTATAAACGGTTCTCTCCAAACCCTCT |
| Tgpd-R | CTTACACGGTCGACTTTCTTTTCTTTACCTGGAAGGGCTGCTGATGGA |
| Padh4-F | TCCATCAGCAGCCCTTCCAGGTAAAGAAAAGAAAGTCGACCGTGTAAG |
| Padh4-R | CTTGGTGTGGATCCAGCCCATTGTGGGGTTCAGATGATGTGTTATTG |
| ZFTF-F | CAATAACACATCATCTGAACCCCACAATGGGCTGGATCCACACCAAG |
| ZFTF-R | CTGCCTTAAAGGATAGAAAGACTTTGTTGATTTATGCATAGCTGATCTCGAAAGATGTCC |
| Tact-F | GGACATCTTTCGAGATCAGCTATGCATAAATCAACAAAGTCTTTCTATCCTTTAAGGCAG |
| Tact-R | GAATACTAATGCCCCCAACTATCCCTATTAAACCTGCCGGAGCTGAAG |
| 18sdown-F | CTTCAGCTCCGGCAGGTTTAATAGGGATAGTTGGGGGCATTAGTATTC |
| 18sdown-R | TACGGAAACCTTGTTACGACTTTTACTTCC |
| Confirm-F | TCGAGATCGATCTCGTTCACAGA |
| Confirm-R | ACTGAATACTAATGCCCCCAACTATCC |
